# Combined bone marrow mesenchymal stem cell-derived nanovesicles and low-level laser therapy potentiate proliferation and osteogenesis of bone marrow mesenchymal stem cells

**DOI:** 10.3389/fbioe.2025.1676777

**Published:** 2025-12-09

**Authors:** Jingwei Zhang, Tonghao Yao, Qi Han, Yongqiang Mo, Dailuo Li, Zhengye Zhang, Lihuang Cui, Zhibin Geng, Weitao He, Jingtao Chen, Xin Liu, Xintao Wang

**Affiliations:** 1 The Second Affiliated Hospital of Harbin Medical University, Harbin Medical University, Harbin, Heilongjiang, China; 2 The First Affiliated Hospital of Shandong Second Medical University, Shandong Second Medical University, Weifang, Shandong, China

**Keywords:** nanovesicles, low-level laser therapy, synergistic bioengineering, bone marrow mesenchymal stem cells, proliferation, osteogenic differentiation

## Abstract

**Background:**

Addressing the persistent challenge of bone defect repair requires innovative bioengineering strategies. Enhancing the biological activity of bone marrow mesenchymal stem cells (BMSCs) is pivotal for effective bone regeneration. This study develops a novel combinatorial bioengineering approach leveraging two distinct biotechnological modalities: low-level laser therapy (LLLT) and bone marrow mesenchymal stem cell-derived nanovesicles (BMSC-NVs). LLLT, a non-invasive biophysical stimulation technique with defined light parameters, is known to prime cellular responses. Concurrently, BMSC-NVs represent an emerging engineered cell-free therapeutic platform with significant promise for tissue regeneration. Thus, we hypothesize that combining LLLT’s direct regulatory effects on BMSCs with the bioactive cargo of BMSC-NVs will synergistically enhance BMSC function. This study presents the first evaluation of the combined impact of LLLT and BMSC-NVs on the proliferation and osteogenic differentiation of rat BMSCs *in vitro*.

**Methods:**

Cell proliferation was quantified using CCK-8 assay, while osteogenic differentiation was assessed through alkaline phosphatase staining, alizarin red staining, and real-time quantitative polymerase chain reaction (osteogenic gene expression).

**Results:**

The LLLT+BMSC-NVs combinatorial strategy effectively enhances BMSC proliferation capacity (as indicated by increased OD values measured via CCK-8 assay), ALP activity, mineralized nodule formation, and upregulation of key osteogenic genes (ALP, RUNX2), showing superior effects on both proliferation and osteogenic differentiation compared to individual LLLT or BMSC-NVs treatments.

**Conclusion:**

This study proposes a novel cell-free therapeutic paradigm by synergistically integrating LLLT with BMSC-NVs, suggesting an effective bioengineering strategy for bone defect repair.

## Introduction

Global population growth and accelerated aging have positioned bone defects as a major threat to human health (Yan et al., 2022; Qi et al., 2025). In 2019, there were 178 million new fracture cases globally, with subsequent refractory bone defects remaining a major clinical challenge (Wu et al., 2021). While autologous bone grafting, the current “gold standard” for bone defect treatment, is limited by donor site morbidity, allogeneic or xenogeneic grafts face safety and ethical constraints (Qi et al., 2025). Therefore, designing novel therapeutic strategies targeting key elements in the bone defect repair process represents both a major challenge and a research focus in current studies. Notably, bone marrow mesenchymal stem cells (BMSCs), which mediate the core processes of bone regeneration, have emerged as critical therapeutic targets in innovative bone repair strategies (Gao et al., 2024; Liu et al., 2024).

Low-level laser therapy (LLLT) has attracted considerable interest for its potential in precisely modulating stem cell activity, while also demonstrating significant efficacy as a promising non-invasive biophysical stimulation technique in tissue regeneration-related applications including inflammation regulation, wound repair, and pain relief (Chen et al., 2022; Azizi et al., 2024; Mayahara et al., 2025). Pasternak-Mnich et al. demonstrated that LLLT significantly promotes mesenchymal stem cell proliferation (Pasternak-Mnich et al., 2024). Wang et al. further confirmed that LLLT enhances the activity of periodontal ligament stem cells (Wang et al., 2022). Critically, LLLT’s ability to synergistically enhance stem cell therapies positions it as a key tool in regenerative biotechnology, as evidenced by accelerated wound healing in diabetic rabbits when combined with adipose-derived stem cells (TizMaghz et al., 2025). However, although LLLT can synergistically enhance the therapeutic outcomes of stem cell therapy, the inherent limitations of traditional stem cell therapies based on exogenous stem cell implantation—including immune reactions, tumorigenicity risks, and ethical concerns—remain inadequately addressed, highlighting the urgent need for next-generation cell-free biotherapeutic strategies (Tan et al., 2024).

Stem cell-derived nanovesicles (NVs) prepared via cyclic mechanical extrusion (diameter: 100–150 nm) represent a novel class of nano-biomaterials containing cargo and membrane structures derived from parent stem cells (Cui et al., 2022). These nanovesicles exhibit high morphological and functional similarity to exosomes secreted by stem cells (key extracellular vesicles secreted by stem cells to mediate tissue repair), while offering distinct bioengineering advantages: approximately 100-fold higher production efficiency, avoidance of the aforementioned risks associated with traditional stem cell therapies, and superior cryopreservation capability (Jang et al., 2013; Jiang et al., 2024). Jiang et al. demonstrated that adipose-derived stem cell-engineered NVs combined with 3D scaffolds promote rabbit radius bone repair (Jiang et al., 2024). Lim et al. showed that umbilical cord mesenchymal stem cell-derived NVs enhance BMSC osteogenic differentiation and accelerate murine calvarial bone regeneration (Lim et al., 2020). These findings validate the potential of stem cell-derived NVs as innovative cell-free therapeutics.

Therefore, by integrating LLLT as a stem cell regulatory biotechnology with the emerging potential of nanovesicles-based nanobiotechnology, this study pioneers a novel combinatorial bioengineering strategy for bone defect repair. In this study, we systematically evaluated for the first time the synergistic enhancement of LLLT combined with bone marrow mesenchymal stem cell-derived nanovesicles (BMSC-NVs) on BMSC proliferation and osteogenic differentiation *in vitro*. This combined strategy establishes a synergistic biological regulatory circuit. Specifically, LLLT acts as a biophysical “priming” stimulus that comprehensively enhances the activity and responsiveness of BMSCs to external cues, creating favorable conditions for combination therapy. Meanwhile, BMSC-NVs can further promote the osteogenic differentiation of the primed BMSCs by delivering various bioactive molecules (e.g., proteins, microRNAs) (Pang et al., 2023). The integration of physical priming and biochemical signaling is expected to generate a synergistic stimulatory effect on BMSC function, potentially surpassing the therapeutic outcomes and limitations of individual monotherapies.

## Methods

### Experimental design and grouping

**FIGURE 1:**
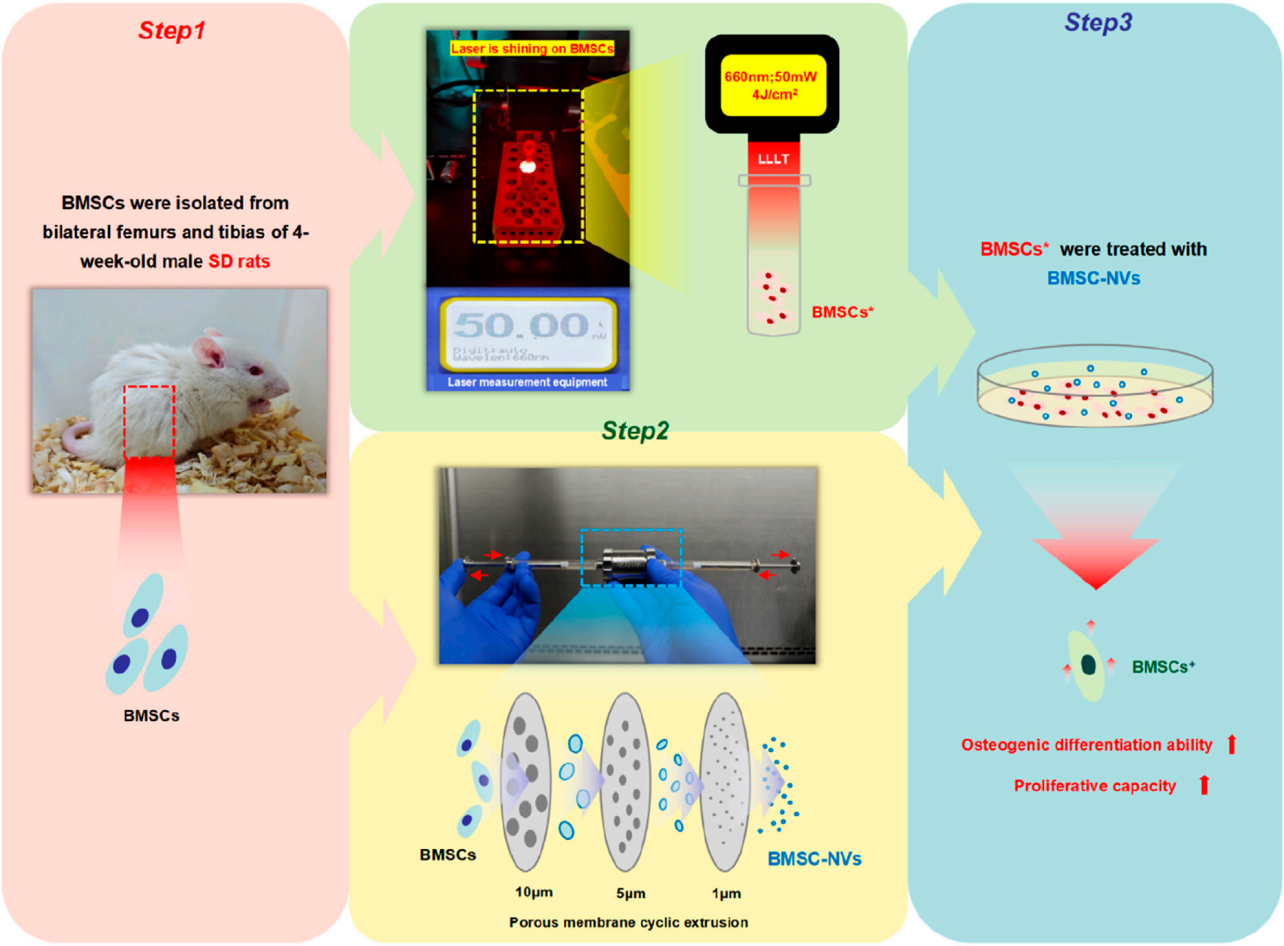
Schematic of experimental design.

The experimental protocol consisted of three steps: (1) isolation of primary BMSCs from Sprague-Dawley (SD) rats; (2) LLLT treatment of BMSCs and preparation of BMSC-NVs; (3) detection of proliferation and osteogenic differentiation (Figure 1). Four experimental groups were established: Control group (BMSCs cultured only), LLLT group (BMSCs treated with LLLT), BMSC-NVs group (BMSCs treated with BMSC-NVs), and LLLT+BMSC-NVs group (BMSCs treated with both LLLT and BMSC-NVs). In all subsequent cellular assays, cells from a single isolation were randomly allocated to different treatment groups. Personnel conducting various experiments and performing analyses remained blinded to group allocations to minimize bias.

### Isolation, culture, and identification of primary BMSCs

The isolation of BMSCs referenced Wang et al.'s methodology (Wang et al., 2025). A total of fifteen 4-week-old male SD rats (95–110 g, SPF grade, purchased from the Laboratory Animal Center of the Second Affiliated Hospital of Harbin Medical University) were euthanized by cervical dislocation performed by trained personnel in strict compliance with the AVMA Guidelines for the Euthanasia of Animals (2020 Edition) which permits conscious cervical dislocation for rodents weighing <200 g. Death was confirmed by absence of corneal reflex, cessation of spontaneous respiration, and cessation of heartbeat, followed by aseptic dissection of bilateral femurs and tibiae. Bone marrow cavities were flushed with complete medium (DMEM/F12 supplemented with 10% fetal bovine serum and 1% penicillin/streptomycin). Cells were centrifuged and seeded in T75 cell culture flasks, then incubated at 37 °C with 5% CO_2_ in a cell incubator. Half-medium replacement was performed at 48 h post-seeding, followed by complete medium replacement every 72 h. Cell morphology and growth status were monitored using an optical microscope. Cells were passaged at a 1:2 ratio when reaching 80%–90% confluency. Third-passage (P3) BMSCs were used for LLLT treatment and various cellular and molecular biology experiments, while fourth-passage (P4) BMSCs were utilized for NVs preparation. Adipogenic differentiation capacity was assessed using Oil Red O staining following the protocol of the Rat BMSC Adipogenic Differentiation Kit (OriCell, RAXMX-90031) on day 10 of adipogenic induction. Osteogenic differentiation capacity was evaluated through Alizarin Red S staining following the protocol of the Rat BMSC Osteogenic Differentiation Kit (OriCell, RAXMX-90021) on day 14 of osteogenic induction. Flow cytometry was further employed to detect the expression levels of BMSC surface markers, including CD29 (BioLegend, 102205), CD90 (BioLegend, 206105), CD45 (BioLegend, 202205), and CD11b (BioLegend, 201805).

### LLLT treatment protocol

All irradiation experiments were conducted in a darkened sterile biosafety cabinet. Laser output power was verified using laser measurement equipment before and after each treatment session (Figure 1). Laser parameters are detailed in Table 1, which were selected based on their validated efficacy in enhancing stem cell function without cytotoxic effects (Si et al., 2022; Wu et al., 2023). BMSCs were digested, centrifuged, and resuspended in aluminum foil-wrapped 2 mL centrifuge tubes fixed on a stable holder. The laser probe was positioned perpendicular to the tube axis at a fixed distance of 10 cm from the tube opening, ensuring the laser beam fully irradiated the bottom of the centrifuge tube. The treated cells were then plated for subsequent experiments.

**TABLE 1:** Laser parameters.

Parameter	Setting value
Wavelength (nm)	660
Energy density (J/cm^2^)	4
Power (mW)	50
Irradiation time (s)	63
Irradiation distance (cm)	10
Number of times	1
Frequency	Continuous

### Preparation of BMSC-NVs

BMSCs (5 × 10^6^ cells) were resuspended in 1 mL PBS and loaded into a liposome extruder. Polycarbonate membranes with pore sizes of 10 μm, 5 μm, and 1 μm were sequentially assembled in the extruder, followed by 10 extrusion cycles for each membrane. The extrudate was centrifuged at 10,000 × g to collect supernatant, followed by purification using 100 kDa ultrafiltration. After the BMSC-NVs were prepared, we conducted a routine quality assessment of their morphology, size and characteristic markers. The morphology was characterized by transmission electron microscopy (TEM). The average particle size of the prepared BMSC-NVs was determined by dynamic light scattering (DLS), with results calculated and exported via Zetasizer software. Western blot analysis was further performed to detect the expression of TSG101 (Abways, CY5985) and CD9 (Abways, CY5337), characteristic markers of BMSC-NVs. BMSC-NVs were stored at −80 °C. The concentration of BMSC-NVs was quantified using the BCA Protein Concentration Assay Kit (Beyotime, P0010S), and a concentration of 80 μg/mL was selected for subsequent experiments.

### Cell proliferation assay

BMSCs were seeded in 96-well plates at 2 × 10^3^ cells/well. After cell adhesion, group-specific treatments were performed. Absorbance at 450 nm was measured at 24 h, 48 h, and 72 h according to the CCK-8 assay kit protocol (Seven, SC119-01).

### Alkaline phosphatase (ALP) and alizarin red staining

Cells from all groups were fixed with 4% paraformaldehyde. ALP staining was performed using protocols specified in the BCIP/NBT Alkaline Phosphatase Color Development Kit (Beyotime, C3206). Alizarin red staining was conducted following the previously described methods. Stained samples were observed under an optical microscope and quantified using ImageJ software.

### Analysis of osteogenic gene expression by real-time quantitative polymerase chain reaction (RT-qPCR)

The mRNA expression levels of osteogenic-related genes ALP and Runt-related transcription factor 2 (RUNX2) were detected via RT-qPCR. After osteogenic stimulation for 3, 7, and 14 days, total RNA was extracted using the Seven RNA key Total RNA Extraction Kit (Seven, SM139-02). cDNA synthesis and PCR amplification were performed according to the protocols of the SevenFast® Two-Step RT&qPCR Kit (Seven, SRQ-01). GAPDH served as the internal reference for RT-qPCR. Relative mRNA expression was calculated by the 2^−ΔΔCt^ method. Primer sequences are listed in Table 2.

**TABLE 2:** Primers for osteogenic factors.

mRNA	Forward (5′–3′)	Reverse (5′–3′)
RUNX2	GCA​CCC​AGC​CCA​TAA​TAG​A	TTGGAGCAAGGAGAACCC
ALP	CAC​GTT​GAC​TGT​GGT​TAC​TGC​TGA	CCT​TGT​AAC​CAG​GCC​CGT​TG
GAPDH	GAGAAGGCTGGGGCTCAC	GTT​GTC​ATG​GAT​GAC​CTT​GGC

### Statistical analysis

Statistical analyses were performed using SPSS software. Normality of data was assessed by Shapiro-Wilk test, while homogeneity of variances was verified via Levene’s test. One-way ANOVA followed by Tukey’s HSD *post hoc* test was applied to evaluate intergroup differences. All experiments included at least three independent replicates. Data are presented as mean ± standard deviation. Statistical significance was defined as *P* < 0.05.

## Results

### Isolation, culture, and characterization of BMSCs

**FIGURE 2:**
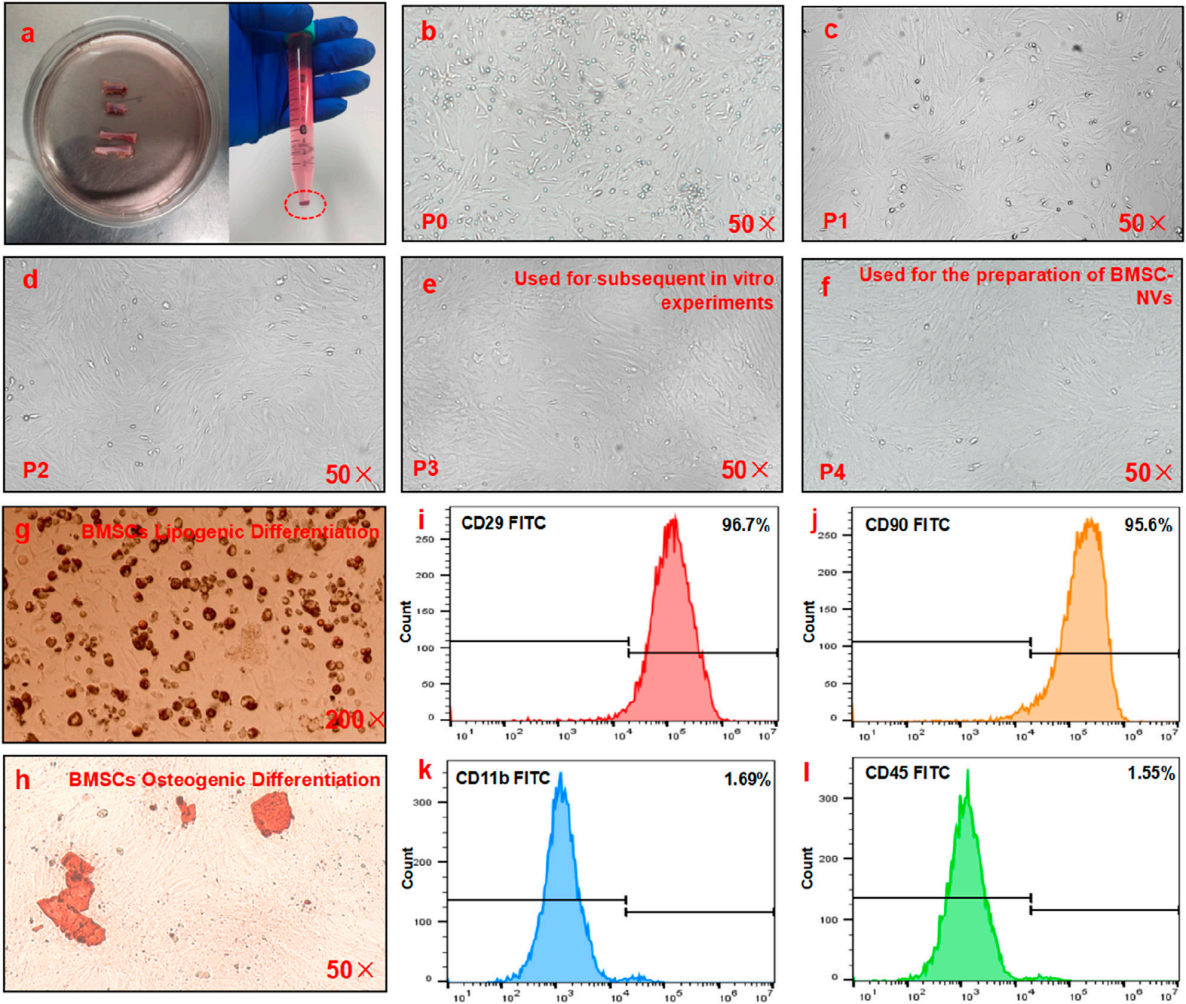
Isolation and characterization of BMSCs. **(a)** The red circles indicate the primary BMSCs isolated from the bilateral tibia and fibula of SD rats. **(b–f)** Optical microscopy of BMSCs at passages 0–4 (P 0–4). **(g)** Oil red O staining of BMSCs. **(h)** Alizarin red staining of BMSCs. **(i–l)** Expression status of surface markers CD29, CD90, CD11b, and CD45 on BMSCs.

Morphological observation under optical microscopy demonstrated that with increasing passage numbers, the purity of isolated primary BMSCs from SD rats progressively improved, accompanied by a gradual reduction in heterogeneous cell populations and the emergence of elongated spindle-shaped morphology (Figures 2a–f). To further validate the identity of BMSCs, their adipogenic and osteogenic differentiation capacities were assessed. Oil Red O staining demonstrated intracellular accumulation of lipid droplets (Figure 2g). Alizarin red staining revealed the formation of mineralized nodules in the extracellular matrix (Figure 2h). Flow cytometric analysis confirmed that the isolated rat BMSCs exhibited positive expression of CD29 (96.7%) and CD90 (95.6%), while negative for CD11b (1.69%) and CD45 (1.55%) (Figures 2i–l).

### Characterization of BMSC-NVs

**FIGURE 3:**
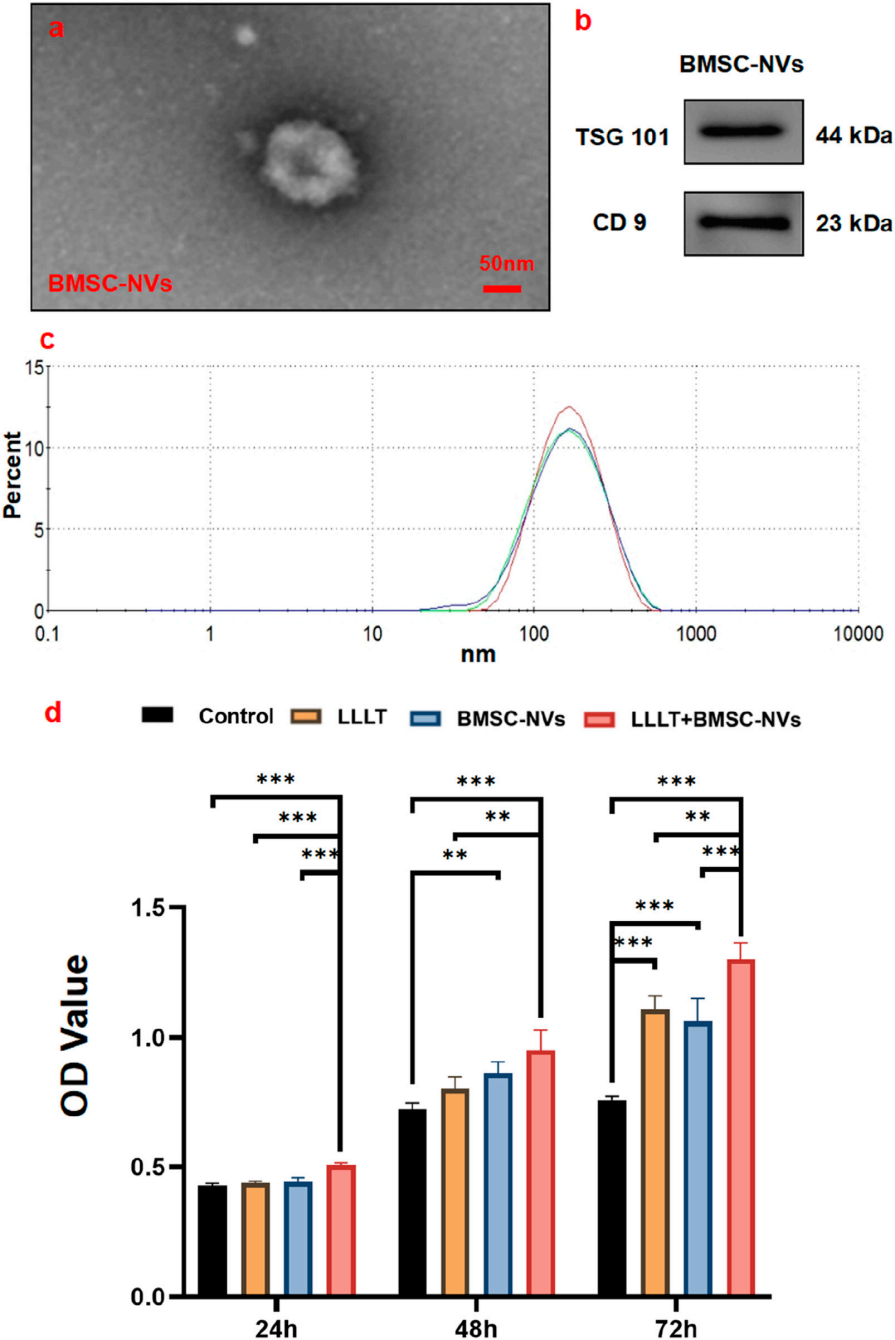
Quality control of BMSC-NVs and synergistic regulation of BMSC proliferation. **(a)** TEM observation of BMSC-NVs. **(b)** Western blot analysis of BMSC-NVs characteristic markers TSG101 and CD9. **(c)** Size distribution of prepared BMSC-NVs determined by DLS. **(d)** Assessment of cell proliferation across groups using CCK-8 assay (*P < 0.05; **P < 0.01; ***P < 0.001).

TEM analysis confirmed that the BMSC-NVs exhibited intact membrane structures with spherical morphology (Figure 3a). Western blot results demonstrated that the NVs characteristic markers TSG101 and CD9 were both positive (Figure 3b). DLS results indicated that the average size of the prepared BMSC-NVs was 138.03 ± 1.03 nm (Figure 3c).

### Cell proliferation

CCK-8 assay revealed a time-dependent increase in BMSC proliferation across all groups. It is worth noting that the LLLT+BMSC-NVs group exhibited the most robust and sustained proliferative advantage, demonstrating significantly higher proliferation levels than all other groups at the 24 and 72 h (Figure 3d). Moreover, both LLLT and BMSC-NVs monotherapies consistently enhanced BMSC growth, showing significantly improved proliferation capacity relative to the Control group at 72 h.

### ALP staining and alizarin red staining with quantitative analysis

**FIGURE 4:**
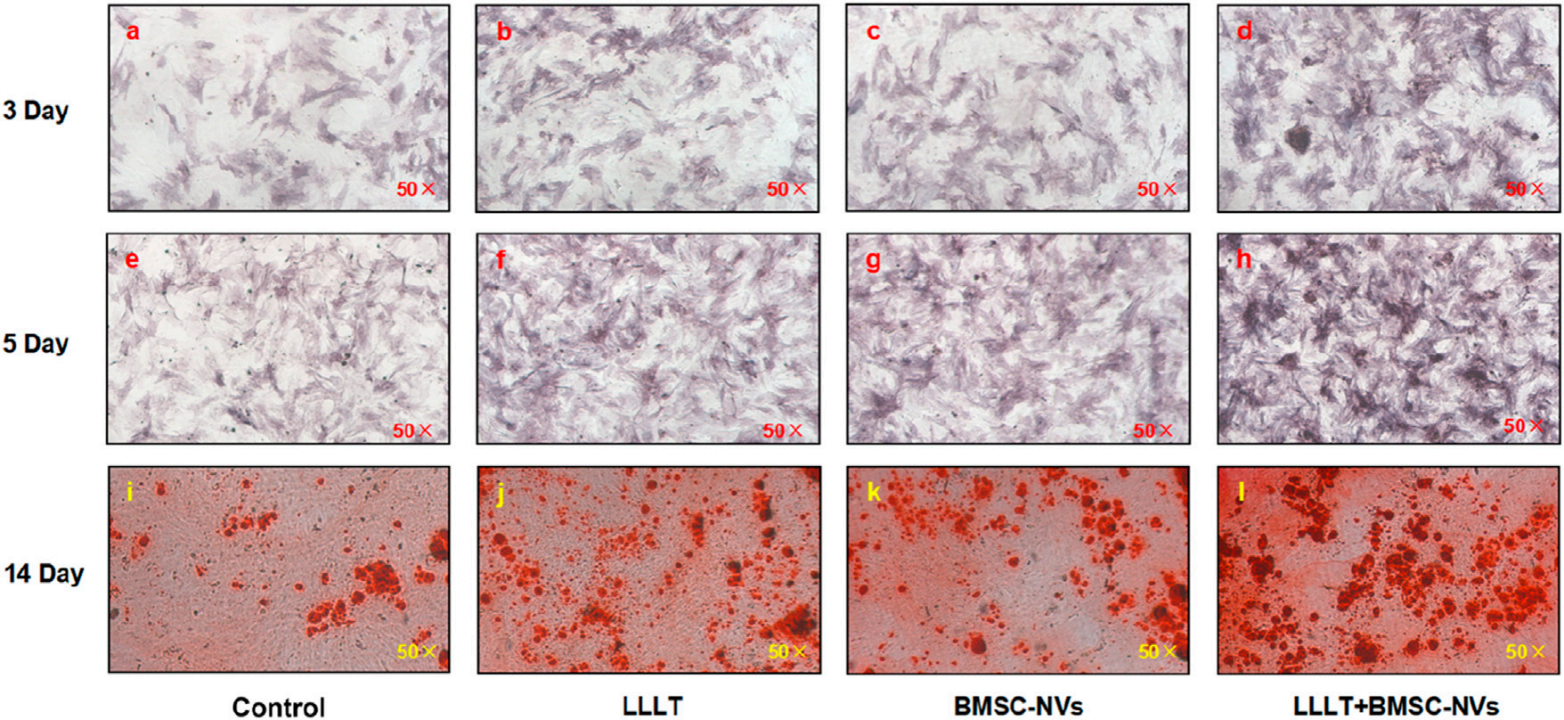
Synergistic regulation of ALP and alizarin red staining in BMSCs by BMSC-NVs and LLLT. **(a–h)** ALP staining results of all groups on day 3 and day 5. **(i–l)** Alizarin red staining results of all groups on day 14.

**FIGURE 5:**
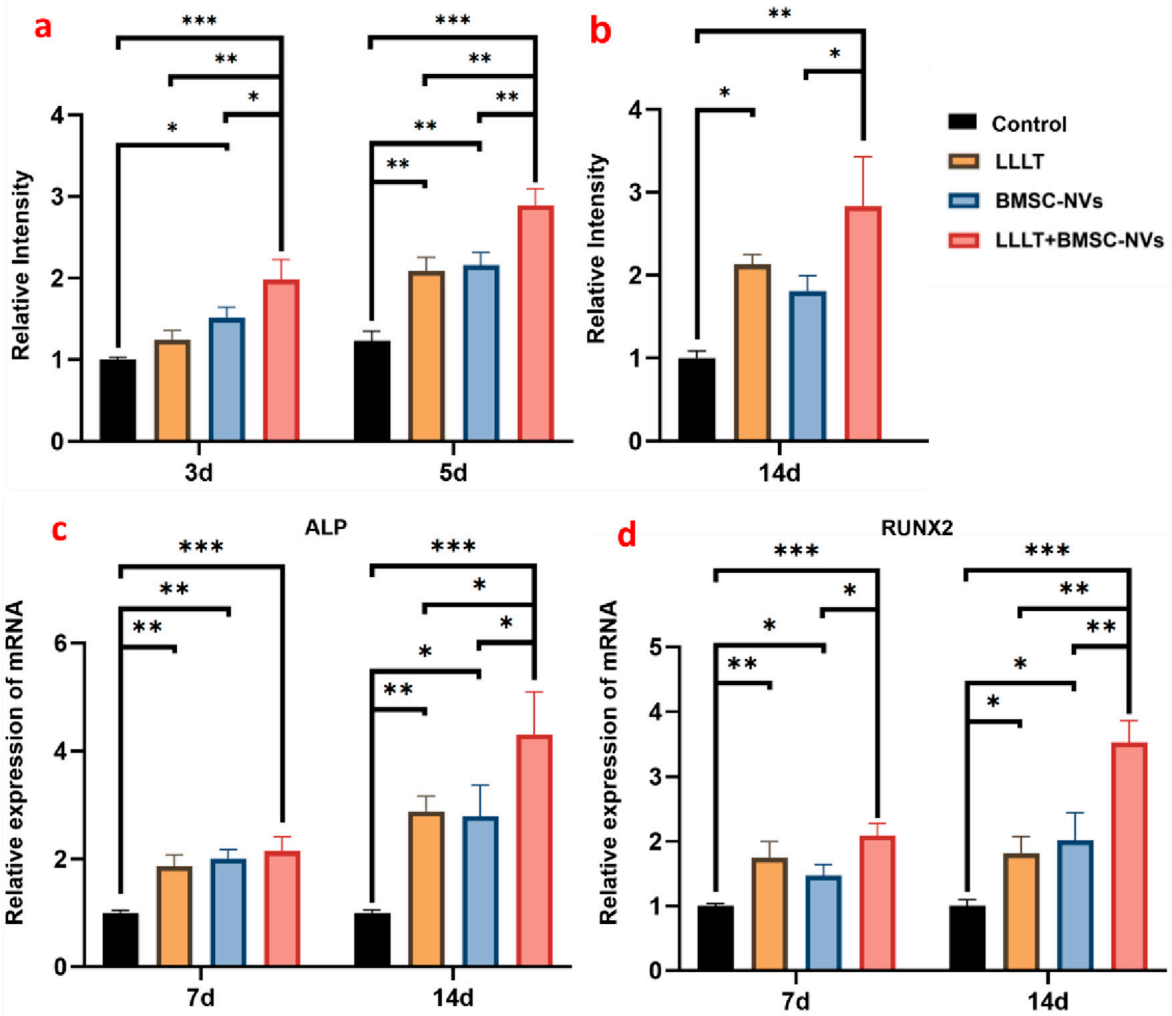
Quantitative analysis of osteogenic differentiation in BMSCs synergistically regulated by BMSC-NVs and LLLT. **(a)** Quantitative analysis of ALP staining. **(b)** Quantitative analysis of alizarin red staining. **(c)** Relative expression of ALP mRNA. **(d)** Relative expression of RUNX2 mRNA (*P < 0.05; **P < 0.01; ***P < 0.001).

Early osteogenic differentiation was assessed through ALP staining and quantitative analysis on days 3 and 5. All treatment groups exhibited a progressive increase in ALP staining intensity over time (Figures 4a–h). Notably, the LLLT+BMSC-NVs group consistently demonstrated the strongest ALP activity, with significantly greater intensity than any other group at both early time points. Individual LLLT and BMSC-NVs treatments also effectively promoted early osteogenesis, as reflected by substantially elevated staining intensity compared to the Control group (Figure 5a). For middle-to-late stage osteogenic differentiation, alizarin red staining and quantification of mineralized nodules on day 14 demonstrated the unequivocal superiority of the combinatorial approach (Figures 4i–l, 5b). The LLLT+BMSC-NVs treatment yielded the most extensive mineralization, displaying the highest staining intensity among groups. In contrast, the Control group formed substantially fewer mineralized nodules with markedly lower staining intensity than all treatment groups (Figure 5b).

### RT-qPCR analysis of osteogenic-related factors

Molecular-level evaluation of osteogenic differentiation was performed via RT-qPCR analysis of ALP and RUNX2 expression on days 7 and 14. All treatment groups showed significant upregulation of both ALP and RUNX2 compared to the Control group (Figures 5c,d). Consistently, the LLLT+BMSC-NVs combination induced the strongest osteogenic response, achieving the highest relative expression levels of both factors. The enhancement in gene expression achieved by this combinatorial strategy was particularly significant on day 14, clearly surpassing the levels induced by either LLLT or BMSC-NVs treatment alone.

## Discussion

This study introduces an innovative multimodal therapeutic strategy based on the integration of two distinct treatment modalities: physical and biological. We synergistically combined LLLT as a physical stimulation mode with BMSC-NVs, which represent a new generation of biological nanotherapy. This integrated approach harnesses the respective advantages of photobiomodulation, which provides early direct stimulation and rapid initiation, and stem cell-based nanotherapy, which exerts a biological signal cascade amplification effect. We constructed a composite bioengineering system and further demonstrated its significant synergistic effect in promoting the proliferation and osteogenic differentiation of BMSCs, thereby establishing a novel cell-free strategy for bone defect repair.

Although numerous studies have systematically explored the biological effects of LLLT on BMSCs, its therapeutic efficacy is highly dependent on multiple parameters such as wavelength, power, and energy density (Berni et al., 2023). While this dependence reflects the advantage of highly tunable modulation in LLLT, it also complicates the selection of optimal treatment parameters. Wu et al. demonstrated that under 660 nm wavelength and 50 mW power, among LLLT irradiation with energy densities ranging from 0 to 4 J/cm^2^, 4 J/cm^2^ not only most strongly promoted the proliferation and osteogenic differentiation of mouse BMSCs but also potentially regulated osteogenic differentiation through BMP2-related signaling pathways (Wu et al., 2012). Similarly, Wang et al. reported that among energy densities from 0 to 8 J/cm^2^, 4 J/cm^2^ most significantly enhanced the osteogenic differentiation of periodontal ligament stem cells, likely via activation of the BMP/Smad signaling pathway (Wang et al., 2022). Since this study focuses on the synergistic effects of LLLT and BMSC-NVs, we directly employed key laser parameters—660 nm wavelength, 50 mW power, and 4 J/cm^2^ energy density—that have been experimentally validated and are commonly used to promote BMSC proliferation and osteogenesis. Output stability was strictly monitored throughout the experiments to ensure reliability. The CCK-8 assay revealed that LLLT significantly promoted BMSC proliferation. Enhanced ALP staining intensity, increased mineralized nodule formation, and upregulated expression of ALP and RUNX2 collectively demonstrated that LLLT under these parameters effectively enhanced the osteogenic differentiation ability of BMSCs. These findings align with previous studies and establish a robust technical foundation for combinatorial therapeutic strategies.

To address the yield limitations of exosomes, Pang et al. efficiently prepared murine BMSC-NVs using the porous membrane cyclic extrusion technique, which effectively promoted BMSC migration and proliferation at a therapeutic concentration of 80 μg/mL (Pang et al., 2023). In this study, rat BMSC-NVs were prepared using the same bioengineering methodology as previously described. Following morphological validation via TEM, DLS, and identification of characteristic markers, the prepared BMSC-NVs were confirmed to meet the required standards. Consequently, a dose of 80 μg/mL BMSC-NVs, consistent with previous literature, was adopted as the standardized therapeutic concentration for subsequent experiments. CCK-8 assay confirmed that BMSC-NVs at this concentration significantly promoted cellular proliferation, consistent with the findings reported by Pang et al. (2023). Additionally, ALP staining, alizarin red staining, and RT-qPCR analysis collectively demonstrated that BMSC-NVs augmented the osteogenic differentiation capacity of BMSCs. Furthermore, compared to the various growth factors currently widely used in clinical bone repair—which are associated with safety and cost concerns such as tumorigenic risk, ectopic bone formation, supraphysiological dosing, and high expense—BMSC-NVs offer a promising alternative (Li et al., 2023). These nanovesicles naturally contain a diverse array of osteogenic factors and miRNAs (Pang et al., 2023). Since they are directly derived from live mesenchymal stem cells, which are inherently involved in bone formation, BMSC-NVs may better recapitulate the multifaceted growth factor microenvironment of physiological bone healing than the application of a single exogenous growth factor. This results in a safer, milder, and more balanced regenerative process. Although some studies have reported that LLLT combined with BMP-2 can also synergistically stimulate bone defect repair, the inherent drawbacks of using a single exogenous growth factor, as described above, remain unresolved (Rosa et al., 2012).

While the individual effects of LLLT and BMSC-NVs on BMSC activity are well-documented, the potential synergistic impact of their combination for bone repair represents a novel and unexplored therapeutic strategy. However, significant breakthroughs have been achieved in antitumor synergistic therapy through the combination of photodynamic therapy, another branch of light therapy, with exosomes or their analogs (Dao et al., 2022). Du et al. demonstrated that combining 532 nm wavelength laser at 100 mW power with engineered exosomes loaded with ferroptosis inducer and photosensitizer effectively induced hepatocellular carcinoma cell ferroptosis (Du et al., 2021). Guo et al. further enhanced breast tumor therapy efficacy by integrating 808 nm wavelength laser at 2 W/cm^2^ with ginger-derived exosome-like nanoparticles encapsulating indocyanine green (Guo et al., 2025). These findings have partially inspired our integration of LLLT with NVs for application in regenerative medicine. Critically, our experimental results demonstrate that the LLLT+BMSC-NVs combinatorial approach exerted significantly superior efficacy in promoting BMSC proliferation and osteogenic differentiation compared to either monotherapy. From a translational perspective, an important implication of our findings is the potential of this synergistic therapeutic strategy to reduce the required therapeutic dosage of BMSC-NVs. Our data indicate that due to the synergistic effects achieved through LLLT combination, the osteogenic outcomes obtained with 80 μg/mL BMSC-NVs were significantly superior to those of monotherapy. This suggests that achieving comparable therapeutic efficacy without LLLT combination would likely require a substantially higher BMSC-NVs dosage, which could potentially introduce medical risks and further increase treatment costs. This synergistic approach, combined with the bioengineering advantages of nanovesicle technology, effectively addresses a critical bottleneck in current cell-free therapeutic strategies - the low yield of natural exosomes - thereby significantly enhancing clinical feasibility.

It is noteworthy that current research on the molecular mechanisms underlying LLLT-mediated osteogenic differentiation remains limited, with its specific regulatory pathways still not fully elucidated. Similarly, studies on stem cell-derived nanovesicles are still in their early stages, with limited investigation into their underlying molecular mechanisms. However, the high morphological and functional similarity between stem cell-derived nanovesicles and stem cell-derived exosomes provides valuable insights for exploring potential synergistic mechanisms. We propose two key mechanisms that may underlie their synergistic effects: First, LLLT may enhance the cellular uptake of BMSC-NVs through regulating endocytosis, thereby amplifying therapeutic efficacy. The study by Svensson et al. demonstrated that ERK1/2 activation is essential for effective exosome uptake and serves as a key target regulating this process (Svensson et al., 2013). Meanwhile, Oliveira et al. showed that LLLT can enhance human osteoblast activity through ERK1/2 activation (Oliveira et al., 2016). Second, LLLT and BMSC-NVs may share common molecular pathways in promoting osteogenesis, which could form the molecular biological basis for their synergistic enhancement. As previously mentioned, LLLT promotes stem cell proliferation and osteogenic differentiation by activating the BMP/Smad signaling pathway. Correspondingly, Zhang et al. demonstrated that BMSC-derived exosomes also promote osteogenic differentiation of mouse embryo osteoblast precursor cells through BMP/Smad pathway activation (Zhang et al., 2020).

This study provides the first evidence that the LLLT+BMSC-NVs combinatorial strategy synergistically enhances BMSC proliferation and osteogenic differentiation far beyond individual treatments. While this strategy has been validated *in vitro*, the cells were cultured in a two-dimensional monolayer, which does not fully recapitulate the three-dimensional physiological microenvironment of bone. Additionally, this approach requires further *in vivo* validation and multi-omics mechanistic exploration. Therefore, future work will focus on validating this strategy in critical-sized bone defect models *in vivo* and exploring its efficacy in more physiologically relevant 3D culture systems, thereby advancing this combinatorial bioengineering strategy toward clinical translation.

## Conclusion

This study provides the first demonstration that the combination of LLLT and BMSC-NVs generates a potent synergistic effect, significantly enhancing the proliferation and osteogenic differentiation of rat BMSCs *in vitro*, far exceeding the outcomes of individual treatments. This bone defect repair strategy, leveraging the synergy between physical and biological bimodal therapies, represents a novel and promising bioengineering paradigm. It effectively integrates the advantages of photobiomodulation with nanotechnology-based cell-free therapeutic strategies while further improving overall treatment efficacy, safety, and cost-effectiveness, thereby establishing a solid foundation for future clinical therapeutic development. Building upon this therapeutic approach, future work will focus on implementing engineered BMSC-NVs strategies to achieve rapid bone repair under complex pathological conditions such as osteoporosis, diabetes, and aging-related bone defects.

## Data Availability

The raw data supporting the conclusions of this article will be made available by the authors, without undue reservation.
