# Supplementary material for: Combined bone marrow mesenchymal stem cell-derived nanovesicles and low-level laser therapy potentiate proliferation and osteogenesis of bone marrow mesenchymal stem cells
Source: Front Bioeng Biotechnol. 2025 Dec 9;13:1676777. doi: 10.3389/fbioe.2025.1676777 (PMC12723869; doi:10.3389/fbioe.2025.1676777)
Supplement: Supplementary file 1 [file Table1.docx]

- Entire original gels
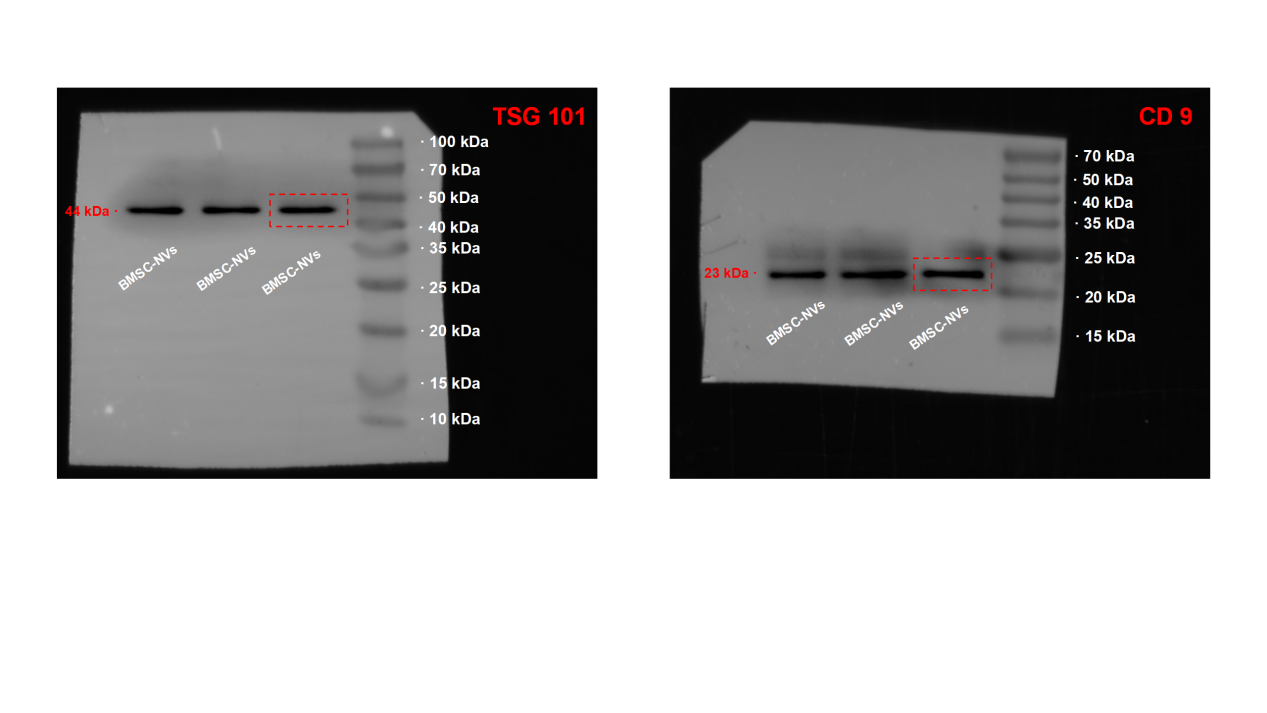
:
  The red-circled area indicates the Western blot protein bands presented in the manuscript.
